# Effect of Heat Curing Method on the Mechanical Strength of Alkali-Activated Slag Mortar after High-Temperature Exposure

**DOI:** 10.3390/ma12111789

**Published:** 2019-06-02

**Authors:** Tai Thanh Tran, Hyuk Kang, Hyug-Moon Kwon

**Affiliations:** 1 Faculty of Civil Engineering, Ho Chi Minh City University of Technology and Education, 1 Vo Van Ngan Street, Thu Duc District, Ho Chi Minh City 700000, Vietnam; thanhtaivlxd@gmail.com; 2 Civil Engineering Department, Yeungnam University, Gyeongsan, Gyeongbuk 712-749, Korea; kh85860@gmail.com

**Keywords:** alkali-activated slag, mechanical strength, high temperature, curing method, brittleness, exposure

## Abstract

The aim of this work was to study the mechanical strength and microstructure changes of alkali-activated slag mortar (AAS mortar) after being heat treated in the temperature range of 200–1000 °C. The AAS mortar was cured in the ambient condition (20 ± 5 °C, 60 ± 5% RH) (Relative humidity: RH) and high temperature condition (80 °C) for 27 days with three different heating regimes: curing in a dry oven, curing in sealed plastic bags, and in a steam environment. The activator for the AAS synthesis was a mixture of sodium silicate solution (water glass) and sodium hydroxide (NaOH) with a SiO_2_/Na_2_O weight ratio of 1, and a dosage of 4% Na_2_O by slag weight. Thermogravimetric analysis (TGA) and scanning electron microscopy (SEM) incorporated with energy-dispersive X-ray spectroscopy (EDX) were used to assess the mortar microstructure change. The results revealed that the curing method significantly affected the mechanical strength of AAS at temperatures lower than 800 °C. The heat treatment at late age of 28 days was more beneficial for compressive strength enhancement in specimens without using heat curing methods.

## 1. Introduction

Alkali-activated ground granulated blast furnace slag has recently attracted strong attention in the literature as a potential alternative to Portland cement [1,2,3]. When used as the main binder, slag is commonly activated by an alkaline agent, such as sodium hydroxide, sodium carbonates, sodium silicates, or other alkalis [4]. Several studies have concluded that the main product of this activation process is generally a low crystalline calcium silicate hydrate (C–S–H) with a low CaO/SiO_2_ ratio [5,6].

The properties of alkali-activated slag (AAS) were concluded to be highly dependent on the type and content of the alkaline activator, and on the curing condition [3,4,7,8,9,10,11,12,13,14]. Investigation of the hydration process of AAS revealed that it was influenced by the sodium dosage and silica modulus M_s_ (a SiO_2_/Na_2_O weight ratio) of the alkaline solution [15]. In addition, AAS cured at an elevated temperature can achieve a high strength at an early age when compared with the ambient cured material [16,17,18]. Elevated-temperature curing greatly accelerates the slag activation process, resulting in the fast development of mechanical strength. In addition, a curing condition with high humidity was reported to be more beneficial than a dry condition since moisture retention is required for hydration [18]. Furthermore, an autoclave curing condition was reported to be appropriate for AAS mortar using an alkaline solution with low Na_2_O concentrations and low weight ratios SiO_2_/Na_2_O (M_s_), whilst steam curing could be suitable for activators that have high M_s_ value [17]. The shrinkage of AAS mortar and concrete was proven to be considerably reduced by curing in an elevated-temperature condition [16,19,20].

AAS was expected to be more durable in an elevated-temperature environment than Portland cement because Ca(OH)_2_ is usually not formed in AAS [21,22,23]. Consequently, the thermal performance of AAS has recently been discussed in many publications. The property of AAS mortar heated to 1200 °C was studied by Zuda et al. [24,25,26]. In these studies, using electrical porcelain as a fine aggregate in AAS mortar was more beneficial than quartz sand with respect to residual strengths at 1200 °C. Rashad et al. [27] discussed the influence of replacing natural sand aggregate with slag on the thermal behavior of AAS mortar in the temperature range of 200–800 °C. AAS mortar attained a higher residual compressive strength with higher slag aggregate content due to the good bond between AAS paste and slag. On the other hand, Guerrieri et al. [28] investigated the property alternation of AAS concrete with the activator, which was a mixture of powdered sodium metasilicate and hydrated lime after exposure to fire treatment of up to 1200 °C. The strength of AAS concrete is reduced at elevated temperatures due to the thermal incompatibility between the AAS paste matrix and the aggregate. The high thermal shrinkage of AAS paste was reported to be the main cause of the high thermal incompatibility of AAS concrete at high temperatures [28]. In addition, Aslani et al. [29] studied the high-temperature performance of heavyweight geopolymer concrete (HWGC), in which the normal-weight coarse aggregates were replaced by heavyweight aggregates (magnetite). However, these studies were conducted on AAS mortar specimens under the same curing condition. Indeed, there are few published studies that examine the effect of curing method on the thermal performance of AAS. Türker et al. [30] carried out high-temperature exposure testing on AAS mortar samples with two different curing methods: ambient curing (specimens were stored in the laboratory condition for 28 days) and high-temperature curing (specimens were cured in a dry oven at a temperature of 60 °C for 6 h and then stored in the laboratory condition for 28 days). Compared with the unexposed specimens, the compressive strength of mortar samples cured in the ambient condition increased by approximately 20% at 200 °C, whereas that of heat cured samples was reduced at all exposure temperatures (from 200 to 800 °C). Nevertheless, few studies have examined the elevated-temperature resistance of AAS mortar under the influence of different heat curing regimes. In particular, Chi [10] showed a variation in the residual compressive strength of AAS concrete with three different curing conditions (air condition, saturated limewater, and the condition with a temperature of 60 °C and relative humidity of 80% RH) (Relative humidity: RH) when exposed to the temperature range of 200–800 °C. Chi reported superior durability after exposure to elevated temperatures for AAS concrete cured at a high temperature and humidity. However, no detailed analysis was conducted to explain this result. 

The main purpose of the present study was to describe the mechanical strength behavior of AAS mortar with different heat curing methods after being subjected to high temperatures of up to 1000 °C. Furthermore, the influences of these different curing conditions on the mortar microstructure change due to heat treatment at late age (high-temperature exposure) are also investigated by using thermogravimetric analysis (TGA), scanning electron microscopy (SEM) and energy-dispersive X-ray spectroscopy (EDX). 

## 2. Materials and Methods

### 2.1. Material Characterization

Blast-furnace slag from a commercial supplier in Korea was used as the raw binder. The chemical composition of the slag is given in Table 1. The specific surface area of the slag was 435 m^2^/kg, as determined by using the Blaine method, and its specific gravity was 2.9.

The activator with a silica modulus (M_s_) of 1 was made by dissolving sodium hydroxide pellets (NaOH) in sodium silicate solution (water glass) that contained 65.4% H_2_O by mass and had a SiO_2_/Na_2_O weight ratio of 3.22. The silica sand with a nominal maximum size of 4 mm and a fineness modulus of 2.45 was used in the mortar mixture as fine aggregate.

### 2.2. Mixture Proportion

The mortar mixture had a fine aggregate to slag weight ratio of 2.75 and a water to solid weight ratio of 0.45. The solid weight was the total weight of slag and solid component in the activator. The Na_2_O concentration of the activator was 4% by slag weight.

### 2.3. Experimental Methods

Mortar mixtures were mixed in a 5 L planetary blending machine in accordance with the specifications of ASTM C305 [31]. The mortar specimens were cast in different shaped molds and kept for 24 h in the ambient condition (20 ± 5 °C and 60 ± 5% RH). The 50 mm cube specimens, 40/40/160 mm prismatic specimens, and 8-shaped specimens were used for the compressive strength test, flexural strength test and tensile strength test, respectively. The mortar specimens were then unmolded and cured in the following four different curing regimes for 27 days prior to heat treatment: (a) A specimens were stored in the ambient condition (20 ± 5 °C, 60 ± 5% RH) (A method); (b) H specimens were cured in a dry oven at 80 °C (H method); (c) B specimens were kept in two sealed plastic bags and then cured in the oven at 80 °C (B method); and (d) S specimens were cured in a steam environment at 80 °C (S method) by using a humidity curing cabinet (Heungjin, model HJ-1450, Gimpo-si, Korea).

**Figure 1:**
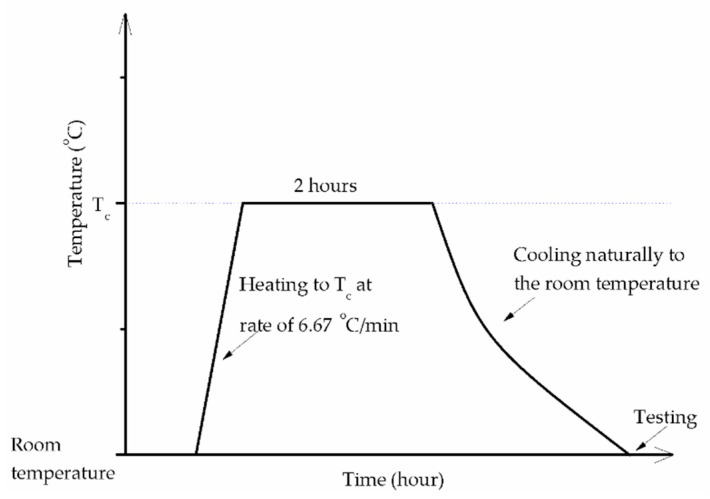
Temperature cycle in the furnace.

After curing, the mortar compressive and flexural strength were tested on the hydraulic press testing machine (Shimadzu, 250 kN, Kyoto, Japan) in accordance with ASTM C109 for compressive strength [32], and ASTM C348 for flexural strength [33]. The tension tester (Heungjin, 5 kN, Gimpo-si, Korea) was used to measure the mortar tensile strength as specified in ASTM C190 [34]. For heat treatment test, the specimens were exposed to high temperatures of 200, 400, 600, 800, and 1000 °C (T_c_) by using a Muffle furnace (Lindberg/Blue, 1300 °C Box Furnace, Waltham, MA, USA) with a heating rate of approximately 6.67 °C/min. The specimens were stored in the furnace at the target temperature (T_c_) for 2 h and then allowed to cool naturally to the laboratory temperature. The temperature cycle in the furnace is depicted in Figure 1.

After cooling to ambient temperature, the mortar specimens were tested to assess the remaining mechanical strength. With reference to some previous publications [23,30,35], selected fragments from the crushed sample after the compressive strength test were soaked in acetone for 72 h for hydration stopping, and then clarified from acetone and finally dried in a vacuum desiccator for 24 h. Some fragments were chosen to use for the SEM (S-4100, Hitachi, Tokyo, Japan) equipped with EDX analysis. Part of the dried samples was crushed into powder for TGA analysis (SDT Q600, New Castle, DE, USA).

## 3. Results and Discussion

### 3.1. Reference Mechanical Strengths

The mechanical strength parameters of the AAS mortar specimens without elevated-temperature treatment at 28 days are given in Table 2. As seen in Table 2, the mechanical strength parameters were greatly influenced by curing conditions. Compared with the A method, the S method greatly increased all mortar mechanical strength parameters, while the flexural strength was reduced under the H and B methods. In addition, the material brittleness was higher when the flexural strength to compressive strength ratio (FS/CS) reduced and the angle of internal friction (Φ) increased [36,37,38]. Consequently, Table 2 shows that the H specimens were the most brittle.

Heat curing treatment at early age greatly enhanced the compressive strength of AAS mortar. For instance, the H, B, and S specimens exhibited strength gains of 44.3%, 44.7%, and 65.9%, respectively, in comparison with the A specimens. However, despite being cured at the same temperature (80 °C), the S specimens achieved approximately 15% greater strength than the H or B specimens. This was due to the quicker escape of water from the sample’s structure when cured in the dry oven and plastic bags. In addition, retained moisture in the steam condition was required for hydration [18].

Table 2 shows that the H and B methods degraded the flexural strength of AAS mortar. For instance, the strength was reduced by 33.8% and 17.6% for the H and B specimens, respectively. Ya-min et al. [39] reported that flexural strength was more easily influenced by the presence of micro-cracks than the compressive strength. The accelerated reaction due to heat treatment at early age caused the large chemical shrinkage and formed micro-cracks in AAS [39]. The specimen structure deteriorated by drying shrinkage due to the water evaporation at 80 °C for 27 days. This can explain why the B and H samples obtained lower flexural strength than the A samples. On the contrary, the S method resulted in a greater strength gain of approximately 64.7% compared with the A specimen. The S method was more effective in increasing flexural strength in comparison with the H or B specimens, possibly due to the limited micro-crack formation in the mortar structure of the S specimens.

The 8-shaped specimens were tested to measure the AAS mortar tensile strength at 28 days. The unexposed B and S specimens possessed similar tensile strengths of approximately twice that of the A and H specimens. Atiş et al. [36] reported that AAS mortar exhibited lower tensile strength when its brittleness increased. However, the H specimens obtained the same tensile strength as the A specimens, despite their higher brittleness.

### 3.2. Residual Compressive Strength

**Figure 2:**
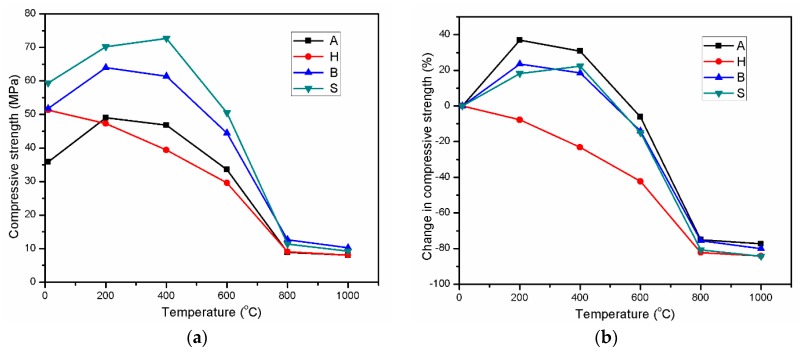
Compressive strength variations (**a**) and percent changes in strength (**b**) of alkali-activated slag (AAS) mortar in the range of 200–1000 °C.

The thermal performance of AAS mortars cured in different methods was evaluated by determining the mechanical strength change after elevated-temperature treatment. The mechanical strength parameters of exposed specimens are shown in Table 3. In addition, the residual compressive strength of specimens after heat treatment at temperatures up to 1000 °C is given in Figure 2a, while Figure 2b illustrates the percent change in compressive strength throughout the range of 200–1000 °C. 

The A and B specimens had the same trend of strength change after high-temperature treatment. At 200 °C, the A and B specimens achieved strength increases of 36.9% and 23.6%, respectively. Raising the temperature to 400 °C reduced this strength increase, but the retained strength remained higher than the reference strengths of 30.7% and 18.5% for specimens A and B, respectively. 

The compressive strength was also enhanced in the S specimens after exposure to 200 and 400 °C. The S specimens exhibited a strength increase of 18.2% and 22.4% at 200 and 400 °C, respectively. Consequently, the S specimens attained the highest strength at 400 °C instead of 200 °C for the A and B specimens.

In contrast to the specimens described above, the H specimens exhibited strength loss at all exposure temperatures. The strength reduction of this mortar was determined to be 7.8%, 23.2%, 42.3%, and 82.3% at exposure to 200, 400, 600, and 800 °C, respectively. The effect of heat treatment on the strength development of AAS has been discussed in many publications [16,18]. In high-temperature curing, the strength of AAS is significantly enhanced at early age. However, the duration of specimens in the laboratory condition prior to heat curing was pointed out to greatly affect the strength gain resulted from heat treatment [16,30]. The compressive strength enhancement of the A specimens at 200 and 400 °C was lower than the strength gain that they could achieve when curing after demolding. On the other hand, the B and S specimens gained higher strength from heat curing after demolding than that attained at 200 and 400 °C. However, the strength gain resulted from the heat treatment at 200 and 400 °C was the highest in the A specimens. In contrast, as shown in Figure 2a, heat treatment at late age did not cause any strength gain in the H specimens. The aforementioned strength enhancement (at 200 and 400 °C) was likely caused by the further hydration acceleration due to heat effect at late age. The results above reveal that the hydration enhancement at late age could be related to the availability of retained water in the mortar structure. The further hydration due to heat treatment at late age was eased with the presence of water in the mortar structure. The considerable loss of water during 27 days at 80 °C in a dry oven was one of the main causes for the lack of strength gain of the H specimens when exposed to 200 and 400 °C.

Raising the temperature beyond 400 °C led to a huge loss of compressive strength for all AAS mortars with the residual strengths converged at 800 °C (Figure 2). The slope of the strength reduction line between 400 and 800 °C was the highest in the S specimens and the lowest in the H specimens. Despite being subjected to elevated temperatures, the S specimens achieved the highest remaining strength and the H specimens the lowest from 200 to 600 °C.

Figure 2 shows that all four specimens exhibited the same remarkable trend of compressive strength change in the temperature range of 800–1000 °C. Increasing the exposure temperature to 1000 °C greatly decreased the strength loss at 800 °C for all specimens. All mortar samples exhibited a slight strength change in this temperature range. In Figure 2a, the compressive strength of the A and H specimens was lower than that of the B and S specimens at all exposure temperatures.

**Figure 3:**
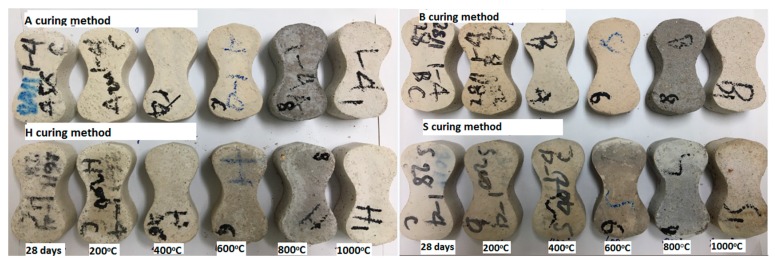
Images of the AAS mortar specimens before and after heat exposure in the range of 200–1000 °C.

The visual observations of hardened mortars before and after exposure to elevated temperatures is exhibited in Figure 3. In the temperature range of 200–800 °C, the white color of the unexposed specimens changed to a darker color under a higher temperature, and then became a lighter color at 1000 °C. Figure 3 shows no sign of spalling in any of the specimens during high-temperature exposure. However, small cracks appeared on the surface of all specimens at 800 and 1000 °C.

### 3.3. Residual Flexural Strength

**Figure 4:**
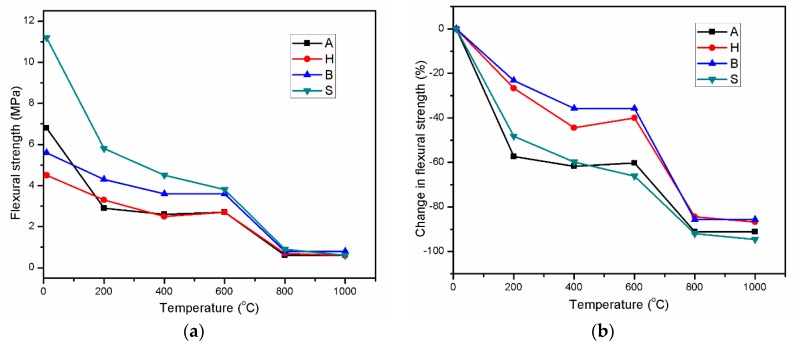
Flexural strength variations (**a**) and percent changes in strength (**b**) of AAS mortar in the range of 200–1000 °C.

Figure 4 illustrates the retained flexural strength and percent change in the strength of AAS mortars in the high-temperature range of 200–1000 °C. Exposure to the high temperature of 1000 °C reduced the flexural strength for all mortars and the strengths converged at 800 °C. Figure 4 shows that the strength change trend of sample H was similar to that of sample B and both exhibited a lower strength loss throughout exposure temperatures to 1000 °C than mortar samples A and S. For instance, exposure to 200 °C reduced the strength by approximately 57.4% and 48.2% for specimens A and S, respectively, compared with 26.7% and 23.2% for specimens H and B, respectively. At 200 °C, the A and S specimens exhibited a drastic strength reduction, and the H and B specimens showed a lower strength drop. The further micro-crack formation from shrinkage may have caused the flexural strength loss of the AAS mortars at elevated temperatures. When subjected to a high temperature of 200 °C, the mortar experienced drying shrinkage due to water evaporation, and chemical shrinkage resulted from further reactions [40]. The formation of cracks due to this mechanism did not occur in the A specimens. However, when exposed to 200 °C, the further rapid reaction and drying shrinkage resulted in significant micro-crack formation and drastically reduced the flexural strength. In the high-temperature condition, AAS experienced large chemical shrinkage due to rapid reaction occurrence, as mentioned by Ya-min et al. [39]. Furthermore, for S specimens, the crack formation could be restrained due to curing in the steam condition. Consequently, the drastic flexural strength reduction in these specimens could be attributed to rapid crack formation in the structure resulting from shrinkage at 200 °C. Increasing the exposure temperature to both 400 and 600 °C alleviated the strength loss for all samples. However, the residual flexural strength of all samples decreased greatly again in the temperature range of 600–800 °C. The residual flexural strength was slightly different at 800 and 1000 °C for all mortar specimens, which is similar to the compressive strength results. Throughout the temperature range from 200 to 1000 °C, the H and B specimens revealed a similar trend in residual strength variation and had a lower strength reduction than the A and S specimens. On the other hand, the A and H specimens exhibited lower flexural strength than the B and S specimens throughout the range of 200–600 °C.

### 3.4. Residual Tensile Strength

**Figure 5:**
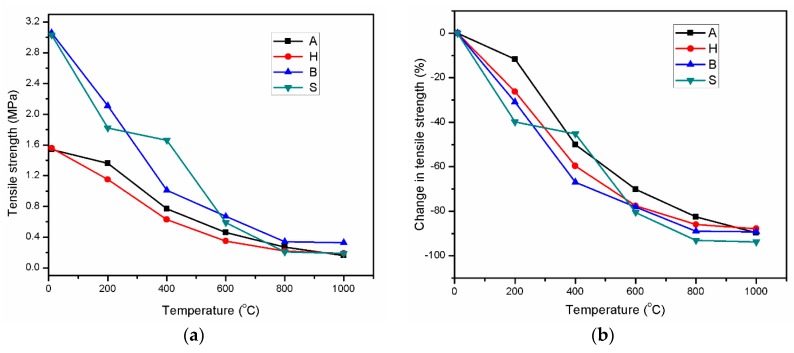
Tensile strength variations (**a**) and percent changes in strength (**b**) of AAS mortar in the range of 200–1000 °C.

Figure 5 depicts the retained tensile strength of AAS mortar in the temperature range of 200–1000 °C. When subjected to high temperatures, the strength of all mortars decreased rapidly at 200 and 400 °C, and exhibited less degradation at higher temperatures. Figure 5 shows that the slope of the residual strength reduction line of samples B and S was greater than that of samples A and H when the temperature was increased to 200 °C, i.e., their strength loss was greater. Previous research pointed out that tensile strength was strongly influenced by the paste matrix-aggregate interfacial transition zone strength [19,41]. Throughout elevated-temperature exposure, the thermal incompatibility between the paste and aggregate degraded the binding ability between the paste and aggregate, while the evolution of pores in the mortar microstructure at high temperatures also decreased the tensile strength [42]. Similar to the compressive strength case, the H specimens exhibited remaining tensile strength lower than that of the other three specimens in 200–600 °C range (Figure 5). More brittle mortar could experience higher shrinkage [36], resulting in higher thermal shrinkage at elevated temperatures [28], and thus, leading to a degradation in mechanical strengths. This result supports the hypothesis of Atiş et al. [36] about the correlation between the shrinkage, brittleness, and tensile strength of AAS mortar. Figure 5 shows that the variation trend of tensile strength between 800 and 1000 °C was similar to that of compressive and flexural strength. Consequently, the influence of curing conditions on mechanical strength change was negligible in the temperature range of 800–1000 °C. On the other hand, the B specimens attained the highest tensile, compressive, and flexural strength at 1000 °C. Similar to the compressive and flexural strength results, the A and H specimens exhibited a low tensile strength in the range of 200–600 °C.

### 3.5. Thermogravimetric Analysis (TGA)

**Figure 6:**
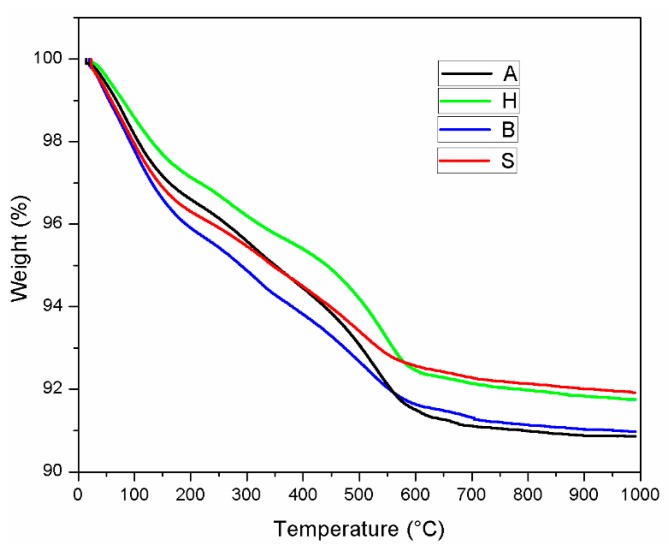
Thermogravimetric analysis (TGA) result of AAS mortar.

Figure 6 presents the TGA curve of the AAS mortar after curing in four regimes. The H sample had the lowest mass reduction before approximately 560 °C. The mass loss before approximately 150 °C is mainly caused by the evaporation of water [30]. The lower mass reduction of H specimens revealed a lower retained water content in its mortar structure, which is unbeneficial for further hydration due to heat treatment at 28 days. In addition, the slope of the mass reduction line in the TGA curve from approximately 450 to 560 °C was steeper in samples A and H than that in samples B and S, indicating their rapid mass reduction in this temperature range.

### 3.6. Microstructural Analysis

**Figure 7:**
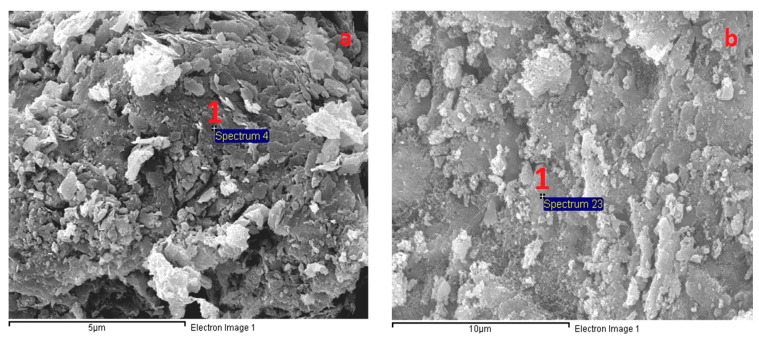

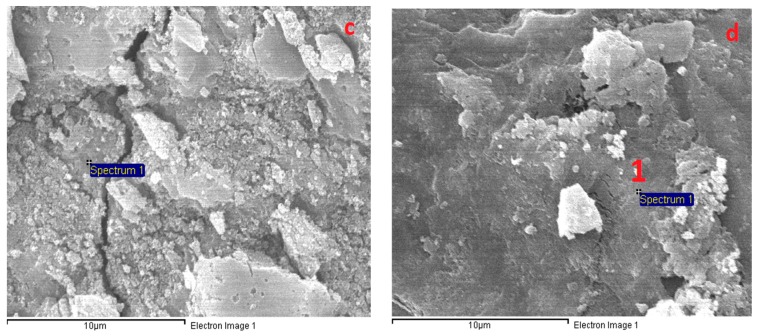
Scanning electron microscopy (SEM) micrographs of AAS mortar after curing by four methods: (**a**) A, (**b**) H, (**c**) B, and (**d**) S.

**Figure 8:**
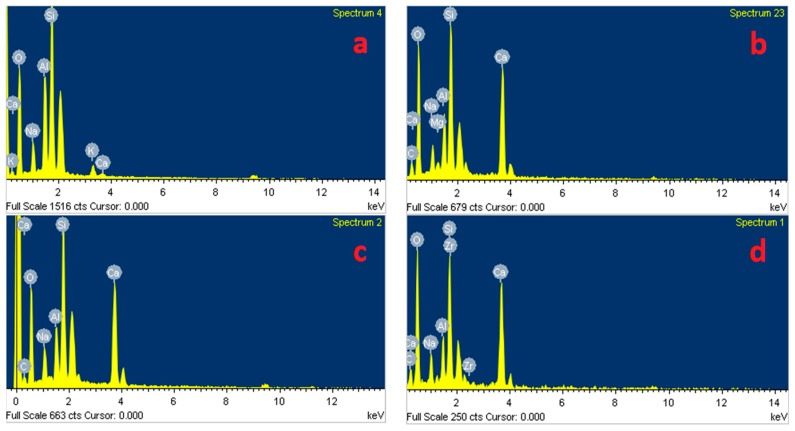
Energy-dispersive X-ray spectroscopy (EDX) analysis results of spots labeled 1 in: (**a**) Figure 7a, (**b**) Figure 7b, (**c**) Figure 7c, and (**d**) Figure 7d.

Figure 7 presents SEM images of the AAS mortar fracture surface with the four different curing methods after casting for 28 days. Figure 7 shows that the A specimens possessed a porous structure with visible unhydrated slag grains. The atomic composition of the reaction product was studied by using EDX. Figure 8a presents the EDX image of the spot marked 1 in Figure 7a where silicon (Si) and aluminum (Al) element intensity was dominant with negligible traces of calcium (Ca). Figure 7 shows that the specimen microstructure became denser following heat curing, which was consistent with the compressive strength enhancement. However, micro-cracks appeared on the surface of the B specimens due to the quick water evaporation, resulting in their low flexural strengths. In addition, both the porous structure and the grain detachment were observed in the H specimens, which may have caused its low flexural and tensile strength. On the contrary, the S specimens exhibited a massive and well-packed microstructure due to the limited water evaporation. The EDX image (Figure 8b–d) shows that the Ca element intensity at the mortar surface (at the point marked 1 in Figure 7b–d) increased remarkably to a dominant level following heat curing. These EDX spectra are also indicative of the existence of C–A–S–H, which was the major hydration product of AAS [4,12]. C–A–S–H in AAS was pointed out to be more amorphous than C–S–H in hydrated Portland cement [43,44]. This clearly demonstrated that the AAS process could be strongly accelerated by heat-treatment curing despite the low Na_2_O concentration (4% by slag weight) of the activator.

**Figure 9:**
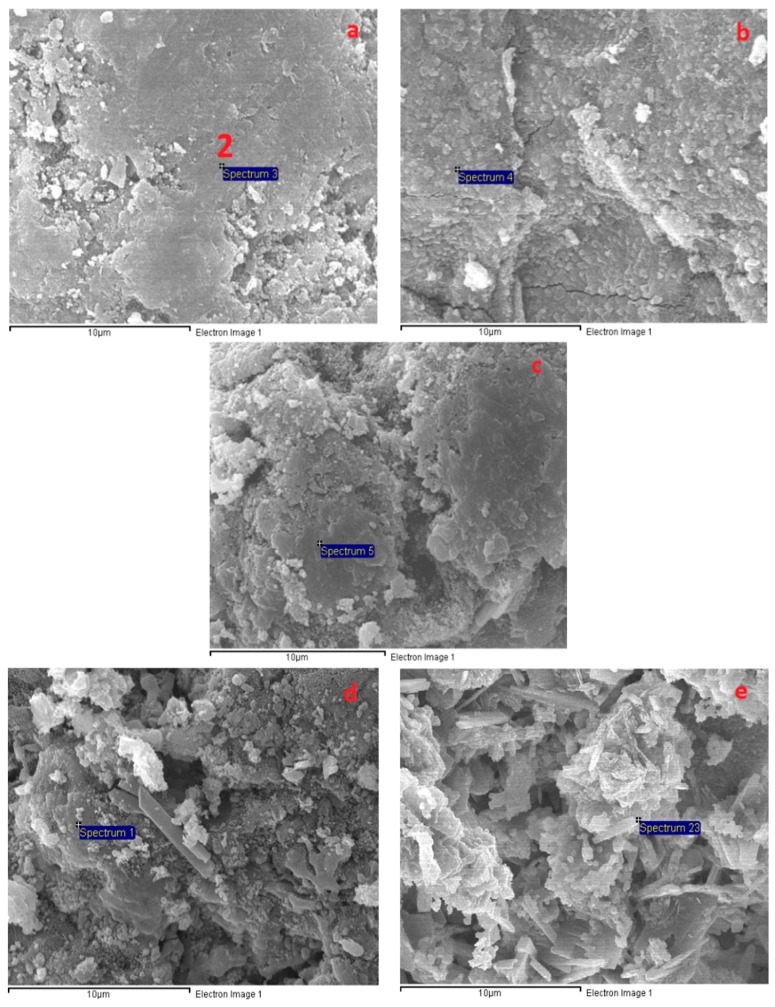
SEM micrographs of the A specimens at different temperatures: (**a**) 200 °C, (**b**) 400 °C, (**c**) 600 °C, (**d**) 800 °C, and (**e**) 1000 °C.

**Figure 10:**
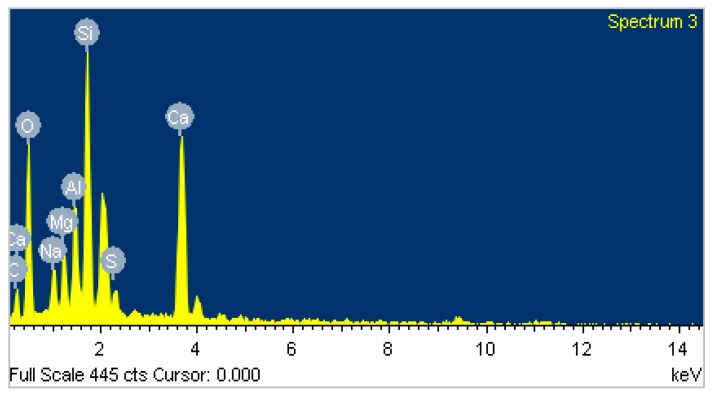
EDX image of spot labeled 2 in Figure 9a.

**Figure 11:**
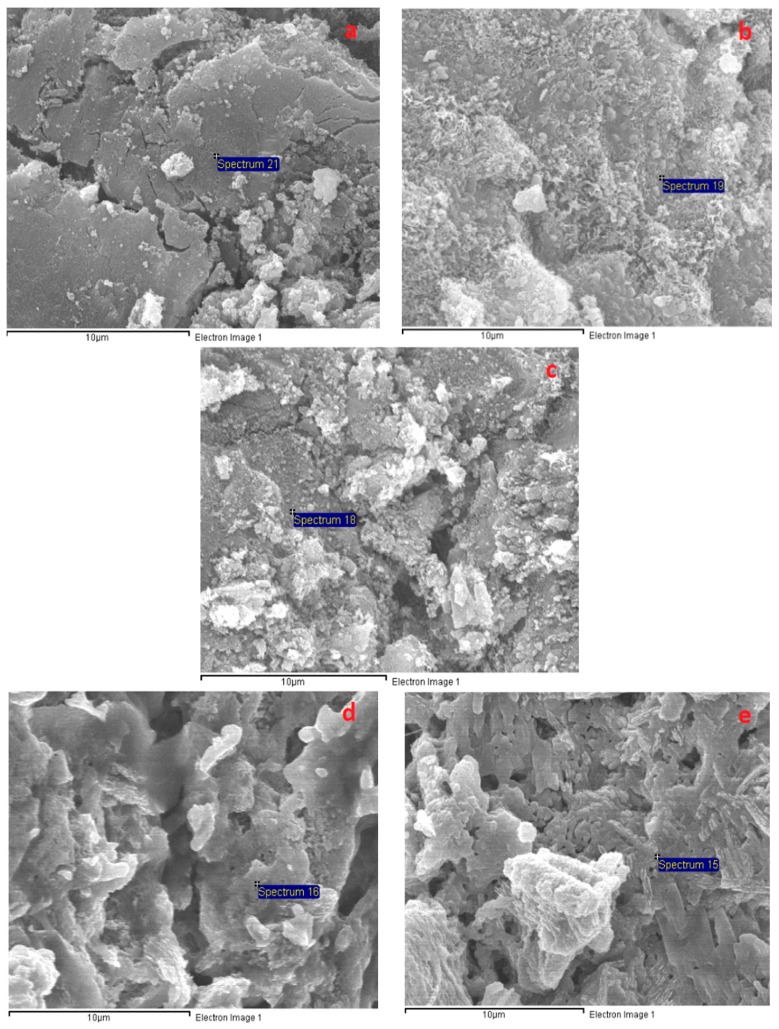
SEM micrographs of the H specimens after exposure to different temperatures: (**a**) 200 °C, (**b**) 400 °C, (**c**) 600 °C, (**d**) 800 °C, and (**e**) 1000 °C.

**Figure 12:**
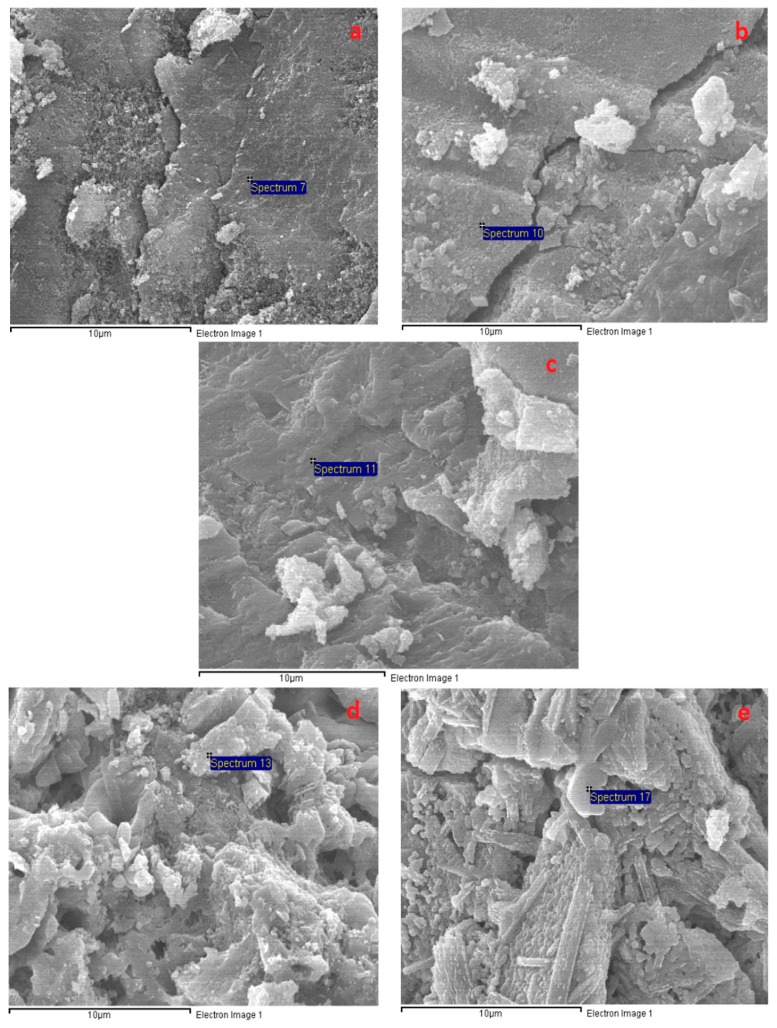
SEM micrographs of the B specimens after exposure to different temperatures: (**a**) 200 °C, (**b**) 400 °C, (**c**) 600 °C, (**d**) 800 °C, and (**e**) 1000 °C.

**Figure 13:**
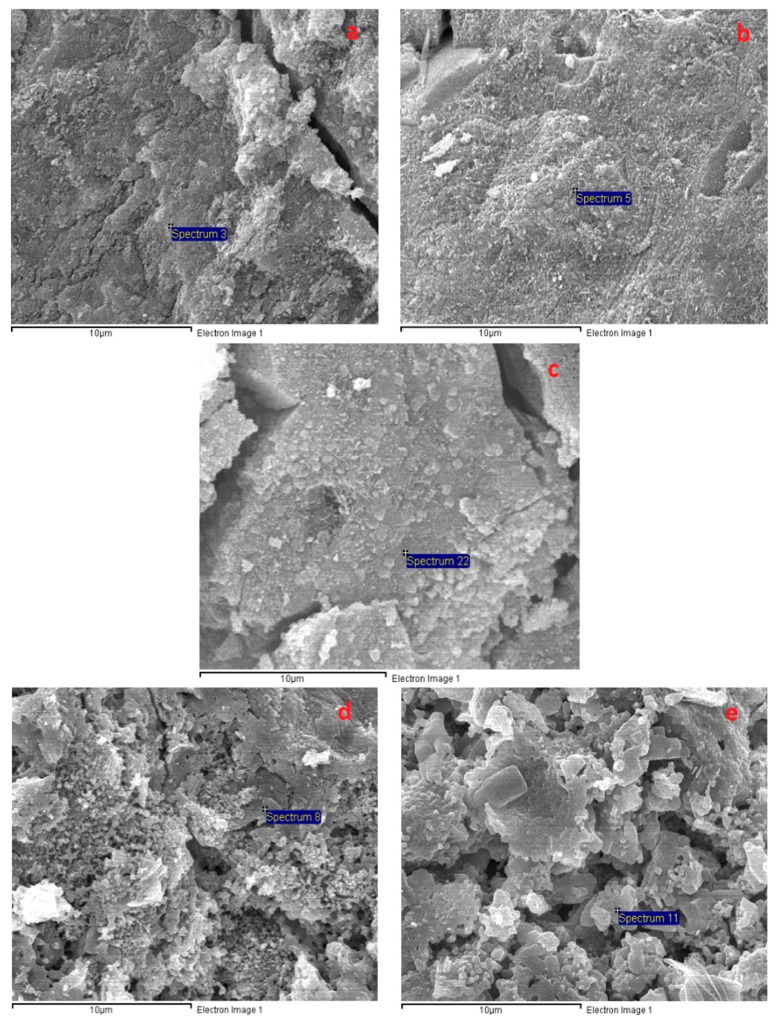
SEM micrographs of the S specimens after exposure to different temperatures: (**a**) 200 °C, (**b**) 400 °C, (**c**) 600 °C, (**d**) 800 °C, and (**e**) 1000 °C.

Figure 9 presents the significant transformation of the A specimen microstructure from 200 to 1000 °C. Figure 9 shows that the porous structure of the A specimens was significantly converted to a denser structure at 200 °C, which may have resulted from further reactions due to the heat treatment effect at 200 °C. In addition, the intensity of calcium (Ca) counts on the A specimen surface (at the spot marked 2 in Figure 9a) increased significantly after exposure at 200 °C (EDX result in Figure 10), revealing the further formation of C–S–H. This is in agreement with the great compressive strength increase and low tensile strength reduction of the A specimens at 200 °C. For the H specimens, large grains and large micro-cracks were detached in the network structure, resulting in the compressive strength drop at 200 °C (Figure 11). The H specimens were clearly the most brittle. Therefore, this mortar exhibited a more damaged structure due to the higher shrinkage at elevated temperatures. Contrary to the A specimens, the surfaces of the S and B specimens presented several fairly large cracks and a slightly less dense area at 200 °C (Figure 12 and Figure 13). This structural transformation contrasts with the compressive strength enhancement of both specimens when exposed at 200 °C.

Figure 9, Figure 11, Figure 12 and Figure 13 show that the mortar structure was significantly degraded after heat treatment in the temperature range of 400–800 °C, causing high strength loss. Crack and pore formation was more intensive in the A and H specimens, resulting from the rapid mass reduction in the temperature range of 450–560 °C (TGA curve). In addition, the network structure of the H sample exhibited significant grain detachment. These results are consistent with the low residual mechanical strengths of the H and A specimens.

As shown in Figure 9, Figure 11, Figure 12 and Figure 13, all mortar samples exhibited a remarkable transformation in network structure in the temperature range of 800–1000 °C. Raising the temperature to 1000 °C healed the cracks and discrete areas in the mortar structure due to sintering. This change in the mortar structure of all mortars considerably reduced the mechanical strength deterioration at 800 °C regardless of the curing method. Figure 12 shows that the B specimens possessed the most condensed microstructure after exposure at 1000 °C. This could explain for the highest mechanical strength of the B specimens when cured at 1000 °C.

## 4. Conclusions

From the experimental results and discussion above, the following conclusions can be drawn:

The heat curing method significantly affected the mechanical strength of AAS mortar at 28 days. The S method induced the greatest strength gain of the four mortar specimens, whereas the H specimens exhibited flexural strength degradation and increased mortar brittleness. 

The A, B, and S specimens exhibited an increased compressive strength at 200 and 400 °C. These 200 and 400 °C heat treatments at 28 days were the most beneficial for compressive strength enhancement in the A specimens. The A specimens achieved a strength gain of 36.9% at 200 °C, compared with 23.7% and 18.2% for the B and S specimens, respectively. By contrast, there was no strength enhancement in the H specimens, which were the most brittle.

AAS mortar specimens with all curing methods exhibited degraded flexural and tensile strength at all exposure temperatures (200–1000 °C). In the range of 200–600 °C, the A and H specimens possessed low residual mechanical strengths due to their damaged structure.

Due to the significant transformation in structure, all AAS mortars exhibited slightly changed mechanical strengths in the temperature range from 800 to 1000 °C regardless of curing methods. The curing method had a negligible effect on the strength change of the mortar in the range of 800–1000 °C. In addition, the B specimens attained the highest residual mechanical strength at 1000 °C.

The SEM and EDX analyses revealed that the porous microstructure of the A specimens was converted to a compact structure with a greatly increased calcium (Ca) amount on the fragment surface after exposure at 200 °C.

Finally, the B and S methods were beneficial for AAS mortar with respect to residual mechanical strength in the range of 200–600 °C. Moreover, the A method was the most effective in enhancing the compressive strength of the mortar at 200 and 400 °C.

## Figures and Tables

**Table 1:** Oxide composition and loss on ignition (LOI) (%) of slag.

Oxide	SiO_2_	CaO	Al_2_O_3_	Fe_2_O_3_	MgO	SO_3_	Na_2_O	K_2_O	LOI
(%)	33.81	41.24	15.19	0.41	5.54	2.51	0.25	0.61	0.18

**Table 2:** Mechanical properties of unexposed specimens at 28 days.

Specimen	Compressive Strength (CS) (MPa)	Flexural Strength (FS) (MPa)	Tensile Strength (TS) (MPa)	FS/CS	Φ * (Radian)
A	35.77	6.82	1.54	0.1907	0.6786
H	51.32	4.5	1.56	0.0877	0.6964
B	51.81	5.62	3.06	0.1085	0.659
S	59.36	11.19	3.03	0.1885	0.6685

* Internal friction angle from Mohr envelope: Φ = 0.5 × arctan((CS − TS)/sqrt(CS × TS)) [36]. (CS, FS, and TS are the compressive, flexural, and tensile strength, respectively).

**Table 3:** Mechanical properties of specimens after high-temperature exposure.

Specimen	Temperature	Compressive Strength (CS) (MPa)	Flexural Strength (FS) (MPa)	Tensile Strength (TS) (MPa)	FS/CS	Φ * (Radian)
A	200	48.97	2.92	1.36	0.0596	0.7005
	400	46.84	2.64	0.77	0.0564	0.7206
	600	33.63	2.71	0.46	0.0806	0.7264
	800	8.92	0.64	0.27	0.0717	0.6966
	1000	8.05	0.61	0.16	0.0758	0.7140
H	200	47.32	3.28	1.15	0.0693	0.7062
	400	39.39	2.52	0.63	0.0640	0.7215
	600	29.57	2.7	0.35	0.0913	0.7306
	800	9.07	0.7	0.22	0.0772	0.7063
	1000	8.07	0.65	0.19	0.0805	0.7075
B	200	64.04	4.28	2.11	0.0668	0.6926
	400	61.44	3.55	1.01	0.0578	0.7206
	600	44.49	3.58	0.67	0.0805	0.7234
	800	12.65	0.8	0.34	0.0632	0.7019
	1000	10.32	0.81	0.33	0.0785	0.6941
S	200	70.17	5.81	1.82	0.0828	0.7035
	400	72.71	4.53	1.66	0.0623	0.7087
	600	50.53	3.82	0.59	0.0756	0.7309
	800	11.44	0.93	0.21	0.0813	0.7168
	1000	9.31	0.58	0.19	0.0623	0.7130

* Internal friction angle from Mohr envelope: Φ = 0.5 × arctan((CS − TS)/sqrt(CS × TS)) [36]. (CS, FS, and TS are the compressive, flexural, and tensile strength, respectively).
